# Supplementary material for: Galectin-9 binds IgM-BCR to regulate B cell signaling
Source: Nat Commun. 2018 Aug 17;9:3288. doi: 10.1038/s41467-018-05771-8 (PMC6098130; doi:10.1038/s41467-018-05771-8)
Supplement: Supplementary file 1 — Supplementary Information [file 41467_2018_5771_MOESM1_ESM.pdf]

Supplementary Information

Galectin-9 binds IgM-BCR to regulate B cell signaling

Anh Cao, Nouf Alluqmani, Fatima Hifza Mohammed Buhari, Laabiah Wasim, Logan K. Smith, Andrew T. Quaile, Michael Shannon, Zaki Hakim, Hossai Furmli, Dylan M. Owen, Alexei Savchenko, and Bebhinn Treanor

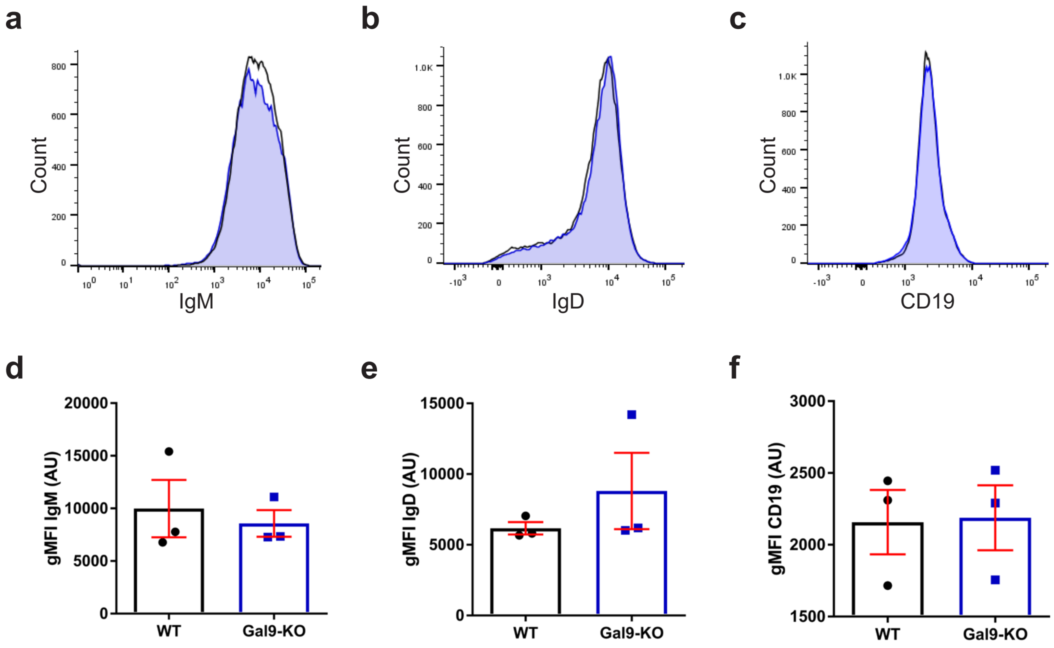

Supplementary Figure 1. Expression of surface proteins in galectin-9 deficient B cells.
(**a-c**) Representative flow cytometry plots of surface IgM (**a**), IgD (**b**), and CD19 (**c**) in WT (black) and Gal9-KO (blue) B cells. Cells were first gated on 7AAD-negative and B220-positive. (**d-f**) Quantification of the geometric mean fluorescence intensity (gMFI) of surface IgM (**d**), IgD (**e**), and CD19 (**f**) in WT (black) and Gal9-KO (blue) B cells. Data representative of three independent experiments. Statistical significance assessed by Wilcoxon matched-pairs signed rank test.

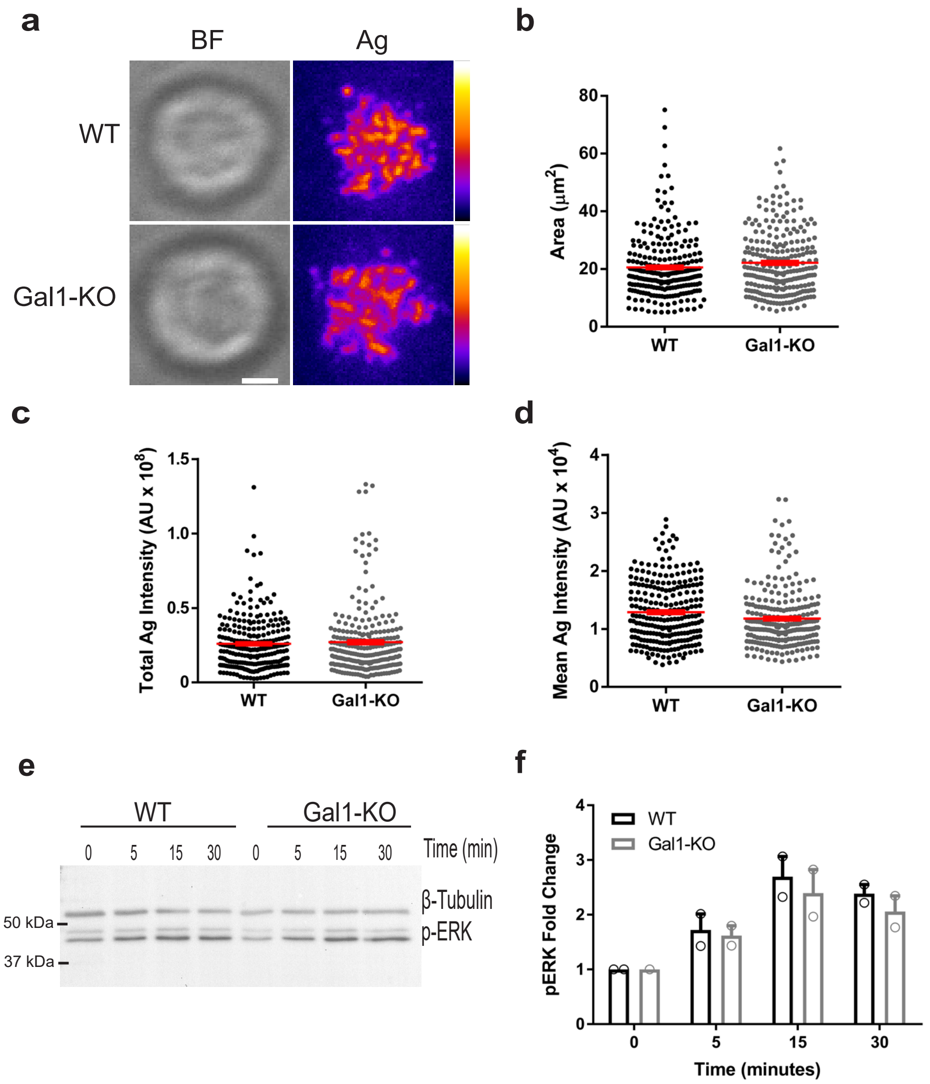

Supplementary Figure 2. Deficiency in galectin-1 does not affect BCR clustering and signaling.
(**a**) Representative images of primary naïve WT (top) and Gal1-KO B cells (bottom) fixed on antigen-containing bilayers after 90 seconds of spreading and imaged by TIRF microscopy. Brightfield (left) and TIRF (right) visualizing antigen mapped to an 8-bit fire colour scale (ImageJ). Scale bar 2 µm. Quantification of (**b**) area of spreading, (**c**) total antigen fluorescence intensity at the cell-bilayer contact, and (**d**) mean intensity of antigen for WT (black circles) and Gal1-KO (grey circles) cells, with the mean ± SEM indicated by the red bar. Statistical significance assessed by Mann-Whitney. Data from three independent experiments were pooled and 250 randomly selected cells were plotted. (**e**) Primary naïve B cells from WT and Gal1-KO mice were settled onto anti-IgM coated plates for the indicated time. Cells were lysed and subjected to SDS-PAGE followed by immunoblotting with anti-phospho ERK1/2 and anti-β tubulin. Data representative of two independent experiments. (**f**) Quantitification of the fold increase in pERK, with the mean ±SEM indicated by bar.

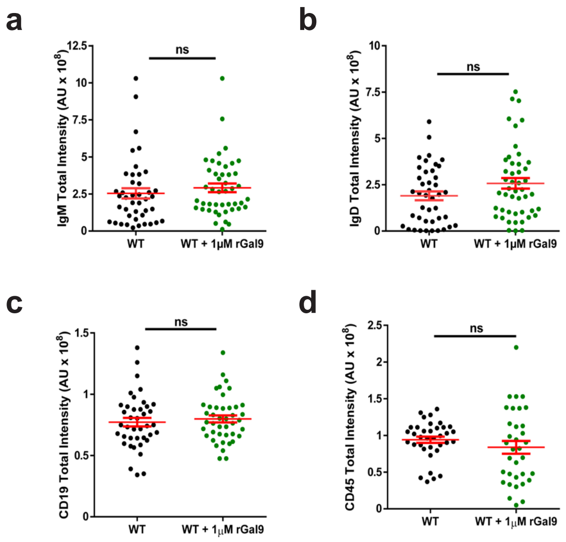

Supplementary Figure 3. Expression of surface proteins in rGal9 treated B cells.
(**a-d**) Surface IgM (**a**), IgD (**b**), CD19 (**c**) and CD45 (**d**) in WT (black) and WT cells treated with 1 μM rGal9 (green) was quantified from z-section confocal microscopy images. Each dot represents one cell, at least 30 cells per condition. Data representative of three independent experiments. Statistical significance assessed by Mann-Whitney, ns = not significant.

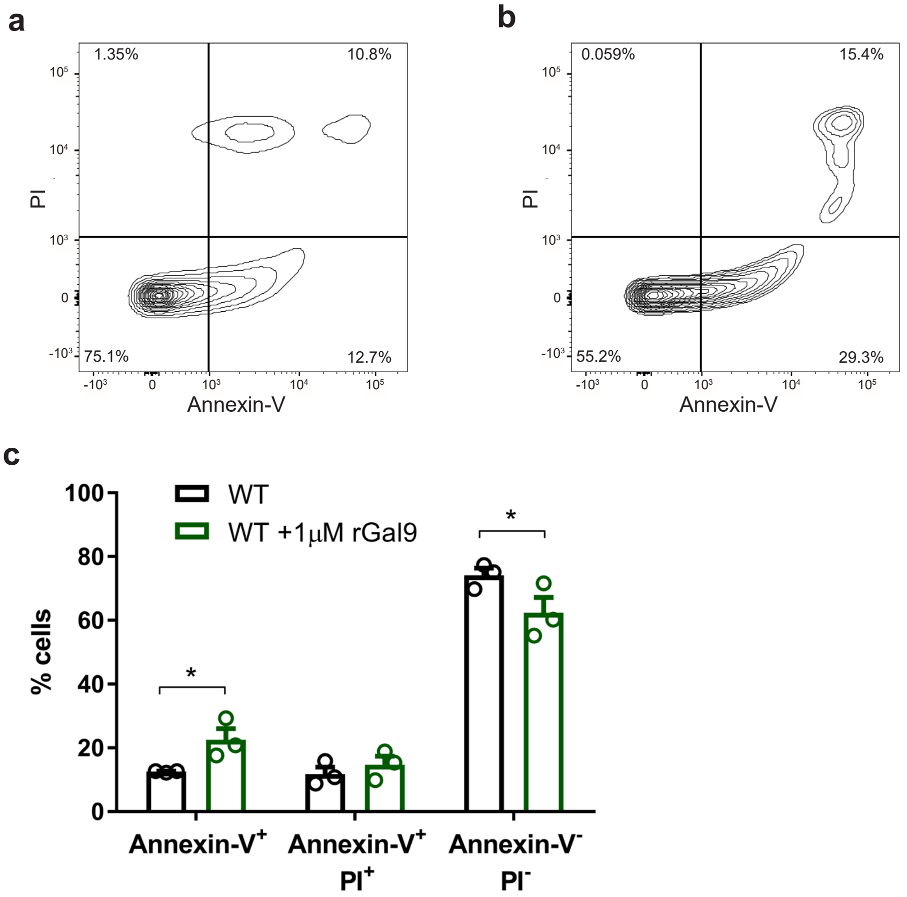

Supplementary Figure 4. Treatment with recombinant galectin-9 increases the number of annexin-V positive cells.
(**a-c**) Representative flow cytometry plots of primary naive B cells from WT (**a**) and WT cells treated with 1 μM rGal9 (**b**) and stained for annexin-V and propidium iodide (PI) and analyzed by flow cytometry. (**c**) Quantification of the percentage of cells positive for annexin-V and PI in WT (black) and WT + 1 μM rGal9 (green). Data representative of three independent experiments. Statistical significance assessed by two way ANOVA followed by Sidak's multiple comparisons. $p<0.05$.

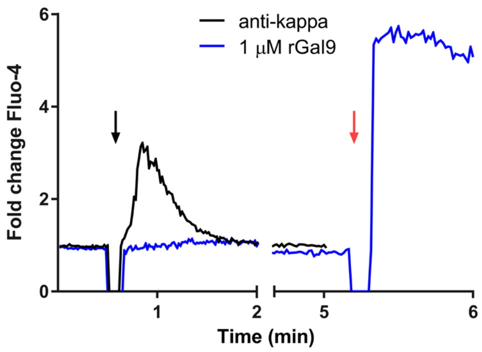

Supplementary Figure 5. Treatment with rGal9 does not induce calcium signaling in B cells.
Intracellular Ca^{2+} flux in primary naive B cells stimulated with anti-kappa (black line) or rGal9 (blue line) measured by flow cytometry. Addition of stimulation (anti-kappa or rGal9) is indicated by black arrow and ionomycin by red arrow. Data are representative of two independent experiments.

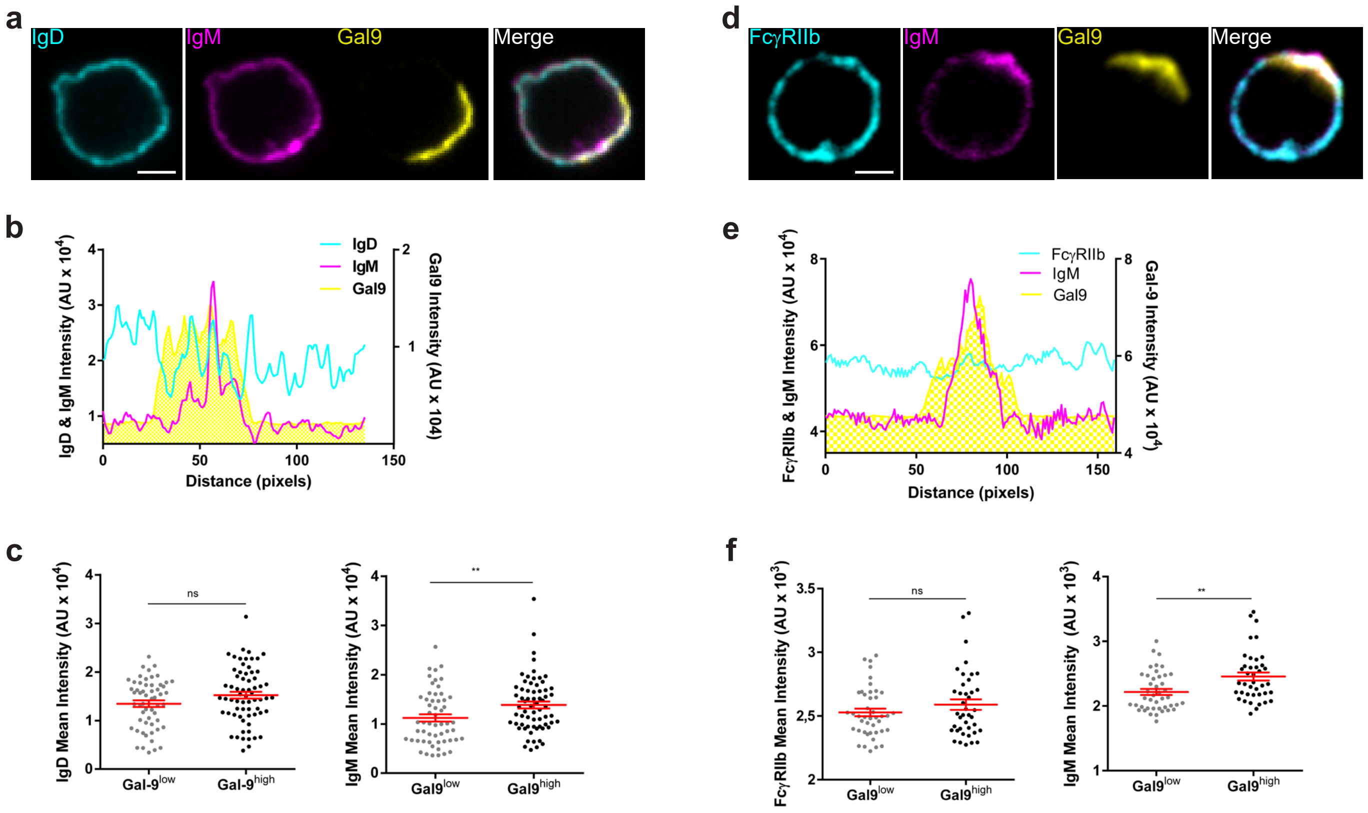

Supplementary Figure 6. The galectin-9 lattice does not increase the molecular density of IgD or FcγRIIb. (**a**) Representative confocal images of WT B cells treated with 1 μM rGal9 and immunostained for IgD (cyan), IgM (magenta), and Gal9 (yellow). (**b**) Fluorescence intensity profile of IgD, IgM, and Gal9 along the cell membrane. (**c**) Mean fluorescence intensity of IgD (left) and IgM (right) in $Gal9^{low}$ and $Gal9^{high}$ regions. (**d**) Representative confocal images of WT B cells treated with 1 μM rGal9 and immunostained for FcγRIIb (cyan), IgM (magenta), and Gal9 (yellow). (**e**) Fluorescence intensity profile of FcγRIIb, IgM, and Gal9 along the cell membrane. (**f**) Mean fluorescence intensity of FcγRIIb (left) and IgM (right) in $Gal9^{low}$ and $Gal9^{high}$ regions. Data are representative of at least three independent experiments. Each dot represents one cell, at least 30 cells per condition. Mean ±SEM indicated by the red bar. Statistical significance assessed by Mann-Whitney, $^{**}p < 0.01$, ns = not significant. Scale bar 2 μm.

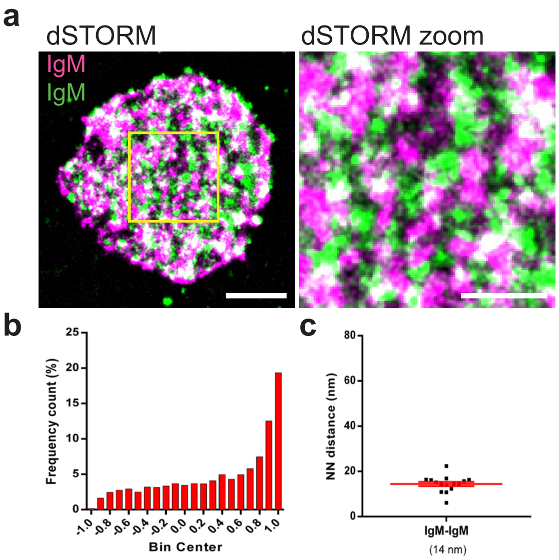

Supplementary Figure 7. Validation of colocalization analysis between Alexa Fluor 647 and Alexa Fluor 488.
(**a**) DSTORM (left), dSTORM zoom (right) images showing surface IgM labelled with Alexa Fluor 647 (magenta) and IgM labelled with Alexa Fluor 488 (green) (left). dSTORM ROI (3 x 3 µm) is outlined in yellow (left) and magnified in dSTORM zoom (right). (**b-c**) Quantification of at least 15 ROIs pooled from 2 independent experiments. (**b**) Coordinate-based colocalization (CBC) histograms of the single-molecule distributions of colocalizations between Alexa Fluor 647 IgM and Alexa Fluor 488 IgM. (**c**) Nearest-neighbor distance (NND) analysis of the data shown in (b). Symbol represents the median NND of all paired single-molecule localizations from one ROI. Colocalization between channels shown in white. Scale bars represent 2 µm and 1 µm (zoom). Lines/errors represent means ± SEM.

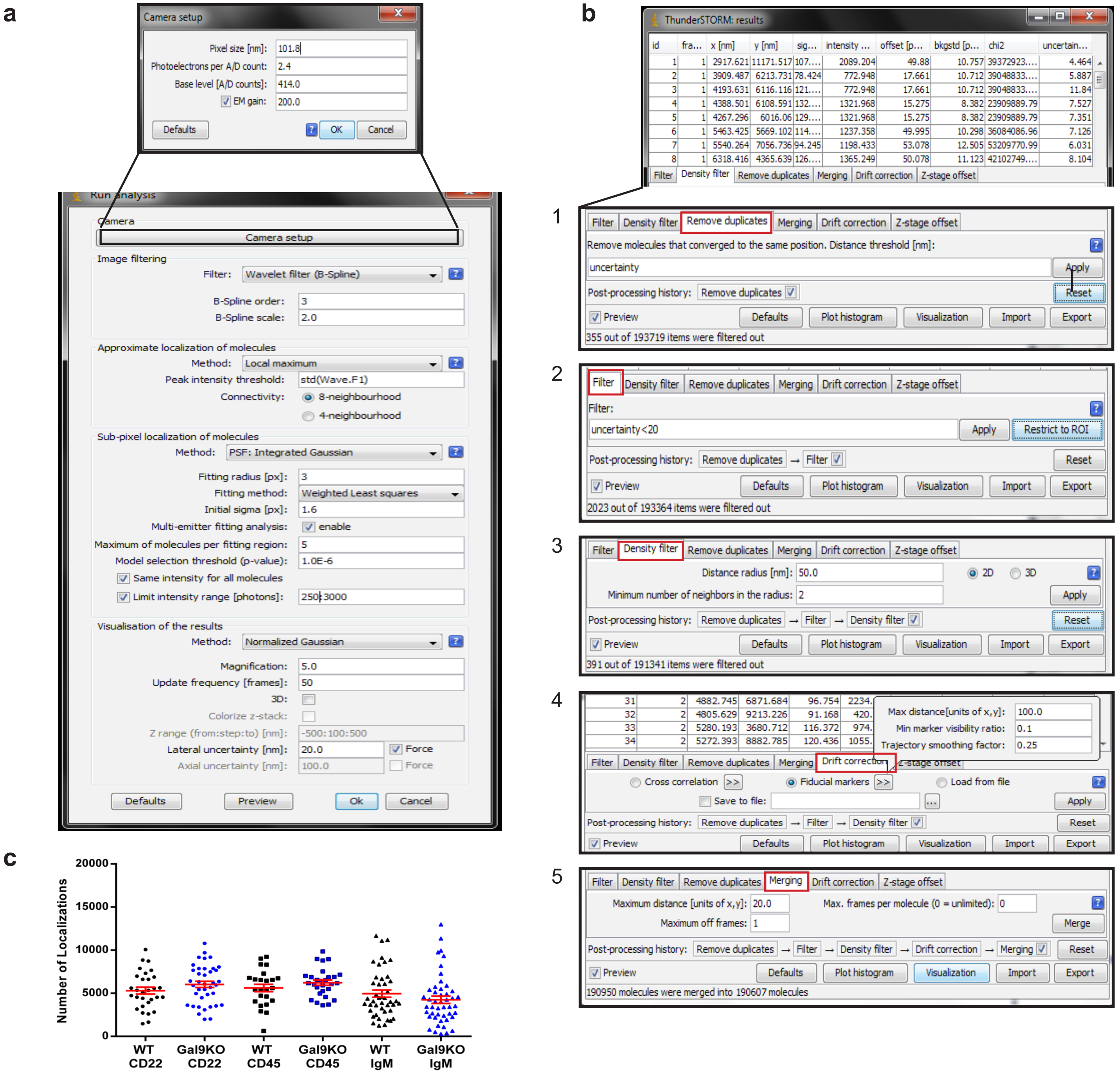

Supplementary Figure 8. dSTORM reconstruction and post-processing parameters used in ThunderSTORM. (**a**) ThunderSTORM image reconstruction parameters and camera settings used for dSTORM image reconstruction from raw stacks. (**b**) Order of ThunderSTORM post-processing parameters used on single molecule localization data for subsequent cluster analysis. (1) Remove duplicates based on localization uncertainty, (2) localizations with uncertainty less than 20 nm are filtered out, (3) isolated localizations are removed with a density filter, (4) sample drift is corrected, and (5) multiple localizations from the same fluorophore are merged together. Single molecule localization data is taken from frames 2000-6000 for cluster analysis by Supercluster. (**c**) The number of localizations used for cluster analysis by Supercluster. Statistical analysis was performed using Kruskal-Wallis test with Dunn's multiple comparison test. Non-significant differences are not indicated.

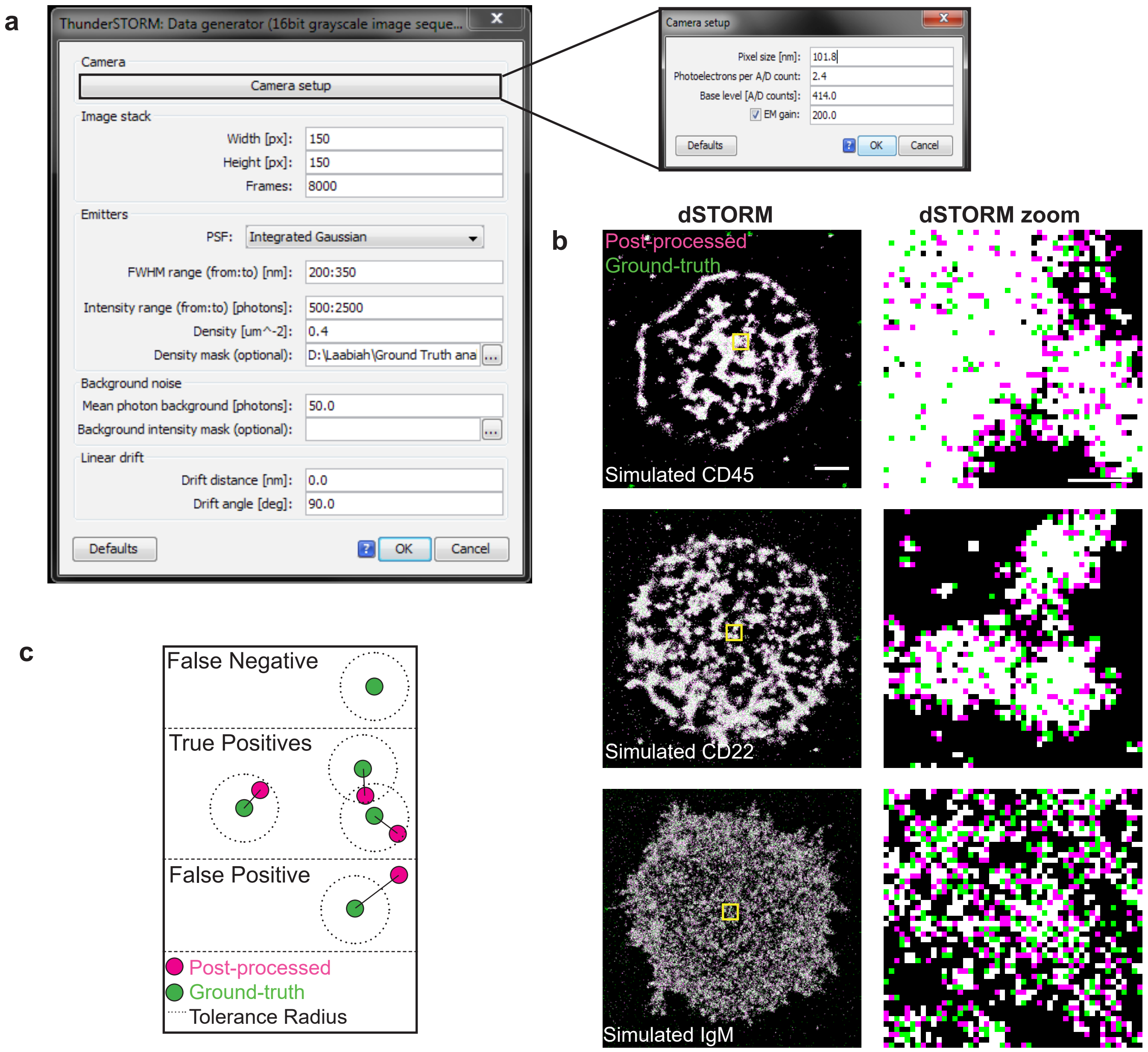

d

	Distance radius [nm]	# of TP	# of FP	# of FN	precision	recall	F1-measure	RMSE lateral [nm]
Simulated CD45 Un-Processed	20	148139	45580	49479	0.765	0.75	0.757	9.174
Simulated CD45 Post-Processed	20	147394	43213	50224	0.773	0.746	0.759	9.163
Simulated CD22 Un-Processed	20	211117	56590	58710	0.789	0.782	0.786	9.111
Simulated CD22 Post-Processed	20	209929	53672	59898	0.796	0.778	0.787	9.1
Simulated IgM Un-Processed	20	142934	29237	29048	0.83	0.831	0.831	8.989
Simulated IgM Post-Processed	20	140598	27230	31384	0.838	0.818	0.828	8.974

Supplementary Figure 9. Performance evaluation of ThunderSTORM analysis and post-processing parameters. (**a**) Parameters used to simulate blinking data in which ground truth positions of single molecule localizations are known. Simulated data stack is reconstructed and post-processed by ThunderSTORM parameters used for real data analysis. The resulting single molecule localization data is compared to ground truth positions for performance evaluation. (**b**) dSTORM images of simulated cells with different cluster organizations (left) and zoomed image of yellow box (right). Ground truth positions (green) and post-processed localizations (magenta) for simulated data. Colocalization between channels shown in white. Scale bars represent 2 μm and 250 nm (zoom). (**c**) Schematic diagram of performance evaluation. True positives (TP) are localizations identified by ThunderSTORM that match ground truth positions within tolerance radius (dotted line). False positives (FP) are localizations identified by ThunderSTORM that do not match ground truth positions. False negatives (FN) are ground truth positions without matching localizations. (**d**) Performance analysis of ThunderSTORM reconstruction and post-processing parameters within tolerance radius of 20nm, comparing un-processed to the corresponding post-processed cell. Perfect matching of localization to ground truth corresponds to precision, recall, and F1 scores of 1.0.

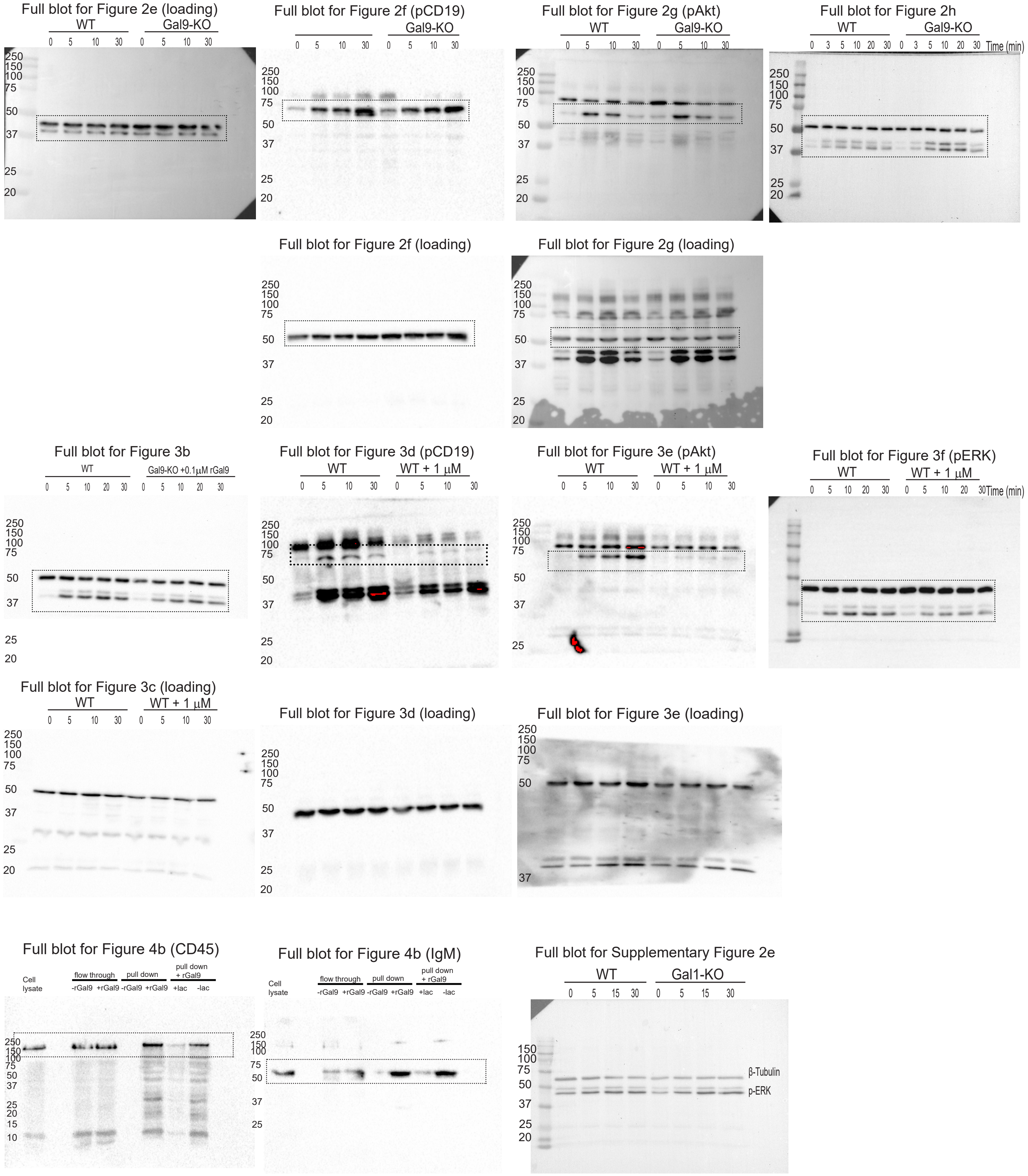

Supplementary Figure 10. Images of uncropped blots.
Images of full blots if cropped in main figure are shown. Area displayed in figure is shown by the black dotted rectangle. Molecular weight makers in kDA are indicated.

Supplementary Methods

Performance evaluation of ThunderSTORM analysis and post-processing parameters. Parameters used in ThunderSTORM for image reconstruction and post processing were evaluated using artificial data where the positions of molecules are computer simulated and therefore are known, also called ground-truth positions. ThunderSTORM's data generator plug-in was used to simulate 8000 frame sequences of artificial blinking data in which positions of molecules were randomly generated within density masks (Supplementary Fig. 9A). Acquired data of CD45, CD22, IgM cells were used for density masks, due to differences in cluster organization, to simulate data that best represented the spectrum of biological data. Camera set-up, intensity range, and density of localization per frame was also kept identical to acquired data. The artificial data stack was subsequently reconstructed and post-processed by ThunderSTORM parameters used for data analysis in this study. DSTORM images of ground truth data and post-processed localization data of simulated cells are shown, with co-localization between the channels in white (Supplementary Fig. 9B). Finally, the single molecule localization data was compared to ground truth positions using ThunderSTORM's built-in performance evaluation plug-in. A tolerance radius of 20nm was chosen for matching localizations to ground truth positions, and this was the pixel size used for reconstructing images.

ThunderSTORM identifies true positives (TP) as localizations that match ground truth positions within the tolerance radius (Supplementary Fig. 9C). False positives (FP) are localizations identified by ThunderSTORM that do not match ground truth positions within the given tolerance radius. False negatives (FN) are ground truth positions that are not associated with any identified localization. The performance evaluation reports multiple statistical measures of recall and

precision that inform on the number of correctly identified, incorrectly identified, and missed localizations. Recall (1) and precision (2) are defined as:

$$(1)\ r = \frac{TP}{TP + FN}$$

$$(2)\ p = \frac{TP}{TP + FP}$$

Recall and precision can be combined as a single measure of performance such as F1 scores, defined as (3).

$$(3)\ F1 = \frac{2pr}{p + r}$$

A F1 value of 1.0 corresponds to a good ratio between precision and recall. Finally, the root mean square error (RMSE) reports on the localization accuracy. RMSE is calculated as the root mean square distance between the ground truth position and the matching localization for all true positive identified. Performance evaluation of simulated cells with analysis and processing parameters used in this study define a localization accuracy of approximately 9 nm between a localization and its ground truth position. ThunderSTORM post-processing parameters result in decreased number of false positives identified while maintaining similar F1 scores and RMSE.
